# Expanding HIV clinical monitoring: the role of CD4, CD8, and CD4/CD8 ratio in predicting non-AIDS events

**DOI:** 10.1016/j.ebiom.2023.104773

**Published:** 2023-08-26

**Authors:** Javier Martínez-Sanz, Jorge Díaz-Álvarez, Marta Rosas, Raquel Ron, José Antonio Iribarren, Enrique Bernal, Félix Gutiérrez, Andrés Ruiz Sancho, Noemi Cabello, Julián Olalla, Santiago Moreno, Sergio Serrano-Villar, CoRIS, Inma Jarrín, David Dalmau, M. Luisa Navarro, M. Isabel González, Federico Garcia, Eva Poveda, Jose Antonio Iribarren, Félix Gutiérrez, Rafael Rubio, Francesc Vidal, Juan Berenguer, Juan González, M. Ángeles Muñoz-Fernández, Inmaculada Jarrín, Cristina Moreno, Marta Rava, Rebeca Izquierdo, M. Ángeles Muñoz-Fernández, Elba Mauleón, Joaquín Portilla, Irene Portilla, Esperanza Merino, Gema García, Iván Agea, José Sánchez-Payá, Juan Carlos Rodríguez, Livia Giner, Sergio Reus, Vicente Boix, Diego Torrus, Verónica Pérez, Julia Portilla, Juan Luís Gómez, Jehovana Hernández, Ana López Lirola, Dácil García, Felicitas Díaz-Flores, M. Mar Alonso, Ricardo Pelazas, M. Remedios Alemán, Víctor Asensi, María Eugenia Rivas Carmenado, Tomás Suarez-Zarracina, Federico Pulido, Rafael Rubio, Otilia Bisbal, M. Asunción Hernando, David Rial, María de Lagarde, Octavio Arce, Adriana Pinto, Laura Bermejo, Mireia Santacreu, Roser Navarro, Candela Gonzalez, Jose Antonio Iribarren, M. José Aramburu, Xabier Camino, Miguel Ángel von Wichmann, Miguel Ángel Goenaga, M. Jesús Bustinduy, Harkaitz Azkune, Maialen Ibarguren, Xabier Kortajarena, Ignacio Álvarez-Rodriguez, Leire Gil, Lourdes Martínez, Félix Gutiérrez, Catalina Robledano, Mar Masiá, Sergio Padilla, Araceli Adsuar, Rafael Pascual, Marta Fernández, Antonio Galiana, José Alberto García, Xavier Barber, Vanessa Agullo, Javier Garcia Abellán, Reyes Pascual, Guillermo Telenti, Lucia Guillén, Ángela Botella, Roberto Muga, Arantza Sanvisens, Daniel Fuster, Juan Berenguer, Isabel Gutierrez, Juan Carlos López, Margarita Ramírez, Belén Padilla, Paloma Gijón, Teresa Aldamiz-Echevarría, Francisco Tejerina, Cristina Diez, Leire Pérez, Chiara Fanciulli, Saray Corral, Francesc Vidal, Anna Martí, Joaquín Peraire, Consuelo Viladés, Montserrat Vargas, Montserrat Olona, Anna Rull, Verónica Alba, Elena Yeregui, Jenifer Masip, Graciano García-Pardo, Frederic Gómez Bertomeu, Sonia Espineira, Marta Montero, Sandra Cuéllar, Marino Blanes, María Tasias, Eva Calabuig, Miguel Salavert, Juan Fernández, Inmaculada Segarra, Juan González-García, Ana Delgado, Francisco Arnalich, José Ramón Arribas, Jose Ignacio Bernardino, Juan Miguel Castro, Luis Escosa, Pedro Herranz, Victor Hontañón, Silvia García-Bujalance, Milagros García, Alicia González-Baeza, M. Luz Martín-Carbonero, Mario Mayoral, M. Jose Mellado, Rafael Esteban, Rocío Montejano, M. Luisa Montes, Victoria Moreno, Ignacio Pérez-Valero, Berta Rodés, Guadalupe Rúa, Talía Sainz, Elena Sendagorta, Eulalia Valencia, Carmen Busca, Joanna Cano, Julen Cardiñanos, Rosa de Miguel, Jose Ramón Blanco, Laura Pérez-Martínez, José Antonio Oteo, Valvanera Ibarra, Luis Metola, Mercedes Sanz, Piedad Arazo, Gloria Sampériz, David Dalmau, Marina Martinez, Angels Jaén, Montse Sanmartí, Mireia Cairó, Javier Martinez-Lacasa, Pablo Velli, Roser Font, Mariona Xercavins, Noemí Alonso, Francesco Aiello, María Rivero, Beatriz Piérola, Maider Goikoetxea, María Gracia, Carlos Ibero, Estela Moreno, Jesús Repáraz, Gemma Navarro, Manel Cervantes Garcia, Sonia Calzado Isbert, Marta Navarro Vilasaro, Belen Lopez Garcia, Ignacio de los Santos, Alejandro de los Santos, Jesús Sanz, Lucio García-Fraile, Enrique Martín, Ildefonso Sánchez-Cerrillo, Marta Calvet, Ana Barrios, Azucena Bautista, Carmen Sáez, Marianela Ciudad, Ángela Gutiérrez, Santiago Moreno, Santos del Campo, José Luis Casado, Fernando Dronda, Ana Moreno, M. Jesús Pérez, Sergio Serrano, Ma Jesús Vivancos, Javier Martínez-Sanz, Alejandro Vallejo, Matilde Sanchez, Jose Antonio Pérez-Molina, José Manuel Hermida, Enrique Bernal, Antonia Alcaraz, Joaquín Bravo, Ángeles Muñoz, Cristina Tomás, Mónica Martínez, M. Carmen Villalba, Federico García, Clara Martínez, José Hernández, Leopoldo Muñoz Medina, Marta Álvarez, Natalia Chueca, David Vinuesa, Adolfo de Salazar, Ana Fuentes, Emilio Guirao, Laura Viñuela, Andrés Ruiz-Sancho, Francisco Anguita, Jorge Del Romero, Montserrat Raposo, Carmen Rodríguez, Teresa Puerta, Juan Carlos Carrió, Mar Vera, Juan Ballesteros, Oskar Ayerdi, Begoña Baza, Eva Orviz, Antonio Antela, Elena Losada, Melchor Riera, María Peñaranda, M. Angels Ribas, Antoni A. Campins, Mercedes Garcia-Gazalla, Francisco J. Fanjul, Javier Murillas, Francisco Homar, Helem H. Vilchez, Luisa Martin, Antoni Payeras, Jesús Santos, María López, Crisitina Gómez, Isabel Viciana, Rosario Palacios, Luis Fernando López-Cortés, Nuria Espinosa, Cristina Roca, Silvia Llaves, Juan Manuel Tiraboschi, Arkaitz Imaz, Ana Karina Silva, María Saumoy, Sofía Catalina Scévola, Adrián Curran, Vicenç Falcó, Jordi Navarro, Joaquin Burgos, Paula Suanzes, Jorge García, Vicente Descalzo, Patricia Álvarez, Bibiana Planas, Marta Sanchiz, Lucía Rodríguez, Julián Olalla, M José Sánchez, Javier Pérez, Alfonso del Arco, Javier de la Torre, José Luis Prada, Onofre Juan Martínez, Lorena Martinez, Francisco Jesús Vera, Josefina García, Begoña Alcaraz, Antonio Jesús Sánchez Guirao, Alvaro Mena, Angeles Castro, Berta Pernas, Pilar Vázquez, Soledad López, Sofía Ibarra, Guillermo García, Josu Mirena, Oscar Luis Ferrero, Josefina López, M. Mar Cámara, Mireia de la Peña, Miriam Lopez, Iñigo Lopez, Itxaso Lombide, Victor Polo, Joana de Miguel, Carlos Galera, Marian Fernández, Helena Albendin, Antonia Castillo, Asunción Iborra, Antonio Moreno, M. Angustias Merlos, Asunción Vidal, Concha Amador, Francisco Pasquau, Concepcion Gil, Jose Tomás Algado, Inés Suarez-García, Eduardo Malmierca, Patricia González-Ruano, M. Pilar Ruiz, José Francisco Pascual, Elena Sáez, Luz Balsalobre, M. Villa López, Mohamed Omar, Carmen Herrero, M. Amparo Gómez, Miguel Alberto de Zarraga, Desiré Pérez, Vicente Estrada, Nieves Sanz, Noemí Cabello, Jorge Vergas García, Maria Jose Núñez, Iñigo Sagastagoitia, Miguel Górgolas, Alfonso Cabello, Beatriz Álvarez, Laura Prieto, Irene Carrillo, José Sanz, Alberto Arranz, Cristina Hernández, María Novella, M. José Galindo, Ana Ferrer, Antonio Rivero Román, Inma Ruíz, Antonio Rivero Juárez, Pedro López, Isabel Machuca, Mario Frias, Ángela Camacho, Ignacio Pérez, Diana Corona, Ignacio Pérez, Diana Corona, Miguel Cervero, Rafael Torres, Juan Antonio Pineda, Pilar Rincón, Juan Macías, Luis Miguel Real, Anais Corma, Marta Fernández, Alejandro Gonzalez-Serna, Eva Poveda, Alexandre Pérez, Luis Morano, Celia Miralles, Antonio Ocampo, Guillermo Pousada, Lucía Patiño, Carlos Dueñas, Sara Gutiérrez, Elena Tapia, Cristina Novoa, Xjoylin Egües, Pablo Telleria

**Affiliations:** a Department of Infectious Diseases, Hospital Ramón y Cajal, IRYCIS, Madrid, Spain; b Hospital Universitario Donostia, Instituto de Investigación BioDonostia, San Sebastián, Spain; c Hospital General Universitario Reina Sofía, Murcia, Spain; d Hospital General Universitario de Elche & Universidad Miguel Hernández, Alicante, Spain; e Hospital Universitario Clínico San Cecilio, Granada, Spain; f Hospital Universitario Clínico San Carlos, Madrid, Spain; g Hospital Costa del Sol, Marbella, Spain; h University of Alcalá, Madrid, Spain; i CIBERINFEC, Instituto de Salud Carlos III, Madrid, Spain

**Keywords:** HIV, Non-AIDS events, Neoplasia, Cardiovascular event, CD4/CD8 ratio

## Abstract

**Background:**

While a low CD4/CD8 ratio during HIV treatment correlates with immunosenescence, its value in identifying patients at an increased risk for clinical events remains unclear.

**Methods:**

We analyzed data from the CoRIS cohort to determine whether CD4 count, CD8 count, and CD4/CD8 ratio at year two of antiretroviral therapy (ART) could predict the risk of serious non-AIDS events (SNAEs) during the next five years. These included major adverse cardiovascular events, non-AIDS-defining malignancies, and non-accidental deaths. We used pooled logistic regression with inverse probability weighting to estimate the survival curves and cumulative risk of clinical events.

**Findings:**

The study included 4625 participants, 83% male, of whom 200 (4.3%) experienced an SNAE during the follow-up period. A CD4/CD8 ratio <0.3 predicted an increased risk of SNAEs during the next five years (OR 1.63, 95% CI 1.03–2.58). The effect was stronger at a CD4/CD8 ratio cut-off of <0.2 (OR 3.09, 95% CI 1.57–6.07). Additionally, low CD4 count at cut-offs of <500 cells/μL predicted an increased risk of clinical events. Among participants with a CD4 count ≥500 cells/μL, a CD8 count ≥1500 cells/μL or a CD4/CD8 ratio <0.4 predicted increased SNAE risk.

**Interpretation:**

Our results support the use of the CD4/CD8 ratio and CD8 count as predictors of clinical progression. Patients with CD4/CD8 ratio <0.3 or CD8 count ≥1500/μL, regardless of their CD4 count, may benefit from closer monitoring and targeted preventive interventions.

**Funding:**

This work was supported by 10.13039/100006301CIBER (CB 2021), 10.13039/501100004587Instituto de Salud Carlos III, Ministerio de Ciencia e Innovación and Unión Europea—NextGenerationEU; by the Spanish AIDS Research Network (RIS) RD16/0025/0001 project as part of the Plan Nacional R + D + I, and cofinanced by 10.13039/501100004587Instituto de Salud Carlos III (ISCIII)- Subdirección General de Evaluación y el 10.13039/501100008530Fondo Europeo de Desarrollo Regional (FEDER), ISCIII projects PI18/00154, PI21/00141, and ERDF, “A way to make Europe”, ICI20/00058.

**Research in context**

**Evidence before this study**

We searched Google Scholar without language restrictions using the terms “HIV”, “non-AIDS events”, “mortality”, “CD8”, and “CD4/CD8 ratio” for articles published through March 6, 2023. We selected studies in which the association of the CD4/CD8 ratio and/or CD8 counts with the incidence of non-AIDS events, including mortality, was reported. The data originated mainly from observational cohort studies, with inconsistent results. The studies presented heterogeneity in the cutoff points studied, the time of measurement, and the methodology employed. Therefore, it remains controversial whether the CD4/CD8 ratio or CD8 count provides additional value to CD4 count in predicting non-AIDS events.

**Added value of this study**

Our study supports the use of a CD4/CD8 ratio cutoff point of <0.3, independent of CD4 count, to identify individuals with HIV at excess risk for cardiovascular events, non-AIDS-defining malignancies, and all-cause mortality. The predictive ability of CD4/CD8 was maintained even in individuals with CD4 count >500 cells/μL, in whom CD4 no longer predicted an increased risk of non-AIDS events. Here, we used landmark analysis and measured our predictor variable at year two from ART initiation. Therefore, the results are easily interpretable and reproducible. Moreover, we reported mid-term events, that are informative for clinicians. Subjects found to be at an increased risk of adverse outcomes may benefit from closer follow-up and targeted preventive interventions.

**Implications of all the available evidence**

The CD4/CD8 ratio has been repeatedly shown to be an indicator of immunosenescence. However, data on its ability to predict non-AIDS events, beyond that of CD4, are inconclusive. The use of an easily interpretable methodology will aid decision-making in routine clinical practice.

## Introduction

People living with HIV (PWH) continue to have a higher risk of mortality and severe clinical events than people without HIV, despite the efficacy of current antiretroviral therapy (ART).^1^, ^2^, ^3^ In this population, CD4 count normalization does not reflect a complete return-to-health state. Persistent residual immune dysfunction contributes to an increased risk of serious non-AIDS events (SNAEs).^4^^,^^5^

The CD4/CD8 ratio has emerged as a useful indicator of immune dysfunction in PWH. It can be easily monitored in routine clinical practice and correlates with markers of immunosenescence and inflammation, which are technically difficult to measure and too variable to be used at an individual level.^6^^,^^7^ PWH with a low CD4/CD8 ratio exhibit increased inflammation and immunosenescence despite successful ART (i.e., having achieved a CD4 count >500 cells/μL).^8^

Several studies have investigated whether the CD4/CD8 ratio predicts the risk of SNAEs or mortality in PWH. However, results are conflicting, with variations in study designs, timing of ratio measurement, ratio thresholds, and endpoints. While some studies have found a significant association between a low CD4/CD8 ratio and an increased risk of SNAEs or mortality,^7^, ^8^, ^9^, ^10^, ^11^, ^12^ others have failed to find this association.^13^^,^^14^ This lack of consensus has led to discrepancies in clinical guidelines regarding the appropriateness of monitoring the CD4/CD8 ratio.^15^^,^^16^ Most evidence linking this marker to clinical outcomes has been generated in retrospective cohort studies, which may be limited by the lack of data on CD8 count, high rates of loss to follow-up, and inability to adjudicate clinical events.^8^^,^^9^^,^^14^^,^^17^^,^^18^ The most informative cut-off values are still unclear, as are the type of events that these markers can predict, and the timing of the measurement relative to ART initiation.^8^^,^^9^^,^^12^^,^^13^^,^^17^^,^^19^ Furthermore, extracting the independent impact of CD8 count is difficult, as their increase may be a homeostatic response to a low CD4 count.^19^

In this study, we aimed to assess whether the CD4/CD8 ratio and CD8 count provide additional prognostic information to CD4 count when measured at year two of ART and to determine the most discriminative thresholds in a large prospective cohort of PWH with long-term follow-up.

## Methods

### Study population

We used data from CoRIS, a prospective multicenter cohort of treatment-naïve adults with HIV, with standardized data collection since 2004. CoRIS collects data from 45 Spanish hospitals. In addition to demographic and clinical data, blood samples are collected and stored in a centralized biobank for the entire cohort. A baseline sample is collected before ART initiation and a follow-up sample is collected annually thereafter. Internal quality controls are performed annually, and 10% of the data are externally audited every two years.^20^ We included individuals with ART initiation up to December 2014 (to allow for a 7-year follow-up) and HIV-RNA <50 copies/mL after two years of ART. We excluded participants with a history of SNAEs, and those with a CD4/CD8 ratio not measured at year two of ART (±3 months).

### Prognostic variables, follow-up, and outcomes

We set the index date (baseline) as the visit occurring two years (±3 months) after ART initiation. Follow-up ended at the earliest loss to follow-up (last date with data update in CoRIS), ART discontinuation for more than 28 days, or administrative end of follow-up.

We explored different prognostic variables at baseline, including (i) reaching a CD4 count above 200, 350, 500 and 750 cells/μL and (ii) reaching a CD4/CD8 ratio above the cut-off values of 0.2, 0.3, 0.4, and 0.5. These thresholds were selected before analysis and were based on previous studies.^8^^,^^9^^,^^12^^,^^13^^,^^17^ Furthermore, we assessed the ability of CD8+ count (at 800, 1000, and 1500 cells/μL cut-offs)^13^^,^^19^ and CD4/CD8 ratio to predict the outcome in the subpopulation of participants with a CD4+ count >500 cells/μL at year two.

The primary outcome was the cumulative incidence of the SNAEs (major adverse cardiovascular event -MACE-, non-AIDS-defining malignancy -NADM-, or non-accidental death as a composite event) during the subsequent five years. The secondary outcomes included the cumulative incidence of (i) MACE, (ii) NADM, and (iii) nonaccidental death. MACE was a composite of nonfatal myocardial infarction, nonfatal stroke, and cardiovascular death.

### Statistical methods

We estimated the five-year risk of experiencing a clinical event using a pooled logistic regression with inverse probability weighting. Each odds ratio required a separate propensity score calculated depending on the categories being compared. Additionally, we computed survival curves by introducing a time-varying intercept, adding the product terms between time and CD4/CD8 ratio. These product terms allowed the hazard ratio to vary over time.

We used inverse probability weighting to control for confounding variables. In addition, we applied inverse probability of censoring weighting (IPCW) for participants who did not remain in follow-up through year five, to address informative censoring. Participants accumulated weights until their event or the end of follow-up, whichever occurred first. Then, standardized weights were created by multiplying the two sets of weights. The estimated weights were truncated at their 99th percentile to prevent outliers from affecting the analyses. The IPCW maintains a constant effective sample size (N = 4625 at ART year 2) for each year of follow-up, transferring the statistical weight of lost or censored participants to those remaining under observation.^21^

Covariates included in the pooled logistic regressions for estimating the weights were age, sex, date of enrolment, mode of HIV transmission, education level, geographical origin, AIDS diagnosis, HIV RNA at diagnosis, type of ART regimen used (a boosted protease inhibitor regimen, a non-nucleoside reverse transcriptase inhibitor, or an integrase inhibitor), and nadir CD4 count. We also included CD8 count at baseline as a covariate in the models for CD4 count. The final models were performed without adding covariates and considering the previously estimated weights. Because the regression coefficients of the covariates are not informative, only the effect estimates for the main variables are reported.

To assess whether the prediction varied for different types of events, we separately conducted analyses on MACE, NADM, and non-accidental death. In a secondary analysis, we performed an *on-treatment* analysis. We assumed that patients who discontinued ART might have remained on treatment had they known of the potential adverse consequences of discontinuing ART. Therefore, censoring due to discontinuation of ART was not considered in these analyses and only censoring due to loss to follow-up was included. We performed all analyses using Stata v.17 (StataCorp LP, College Station, TX, USA).

### Role of funders

The Funders had no role in study design, data collection, data analyses, interpretation, or writing of report.

### Ethics

This study was approved by the Institutional Review Boards of the Carlos III Health Institute located in Madrid, Spain and by the Ethics Committee at University Hospital Ramón y Cajal (ceic.hrc@salud.madrid.org, approval number 225–16). All participants included in CoRIS gave their informed consent. This work has been carried out in compliance with ethical approval.

## Results

We included 4625 participants. The study flowchart is shown in Figure S1. The median age was 37 years (IQR 31–44), 3852 (83%) were male, and the median CD4/CD8 ratio at ART initiation was 0.29 (IQR 0.17, 0.46). Participants who had an event during follow-up showed a higher percentage of intravenous drug use as a risk factor for HIV transmission, lower educational level, lower nadir CD4 count, and lower CD4/CD8 ratio (Table 1).

**Table 1:** Baseline characteristics of the study population.

Baseline^a^ characteristics	Event (n = 200)	No event (n = 4425)
**Age, median (IQR)**	47 (39, 54)	37 (30, 43)
**Sex, male, n (%)**	165 (83)	3687 (83)
**Mode of transmission, n (%)**		
MSM	75 (38)	2534 (57)
Heterosexual	36 (18)	355 (8)
IDU	81 (41)	1396 (32)
Other	8 (4)	140 (3)
**Education level, n (%)**		
None—Primary	49 (25)	658 (15)
Secondary—High school	92 (36)	2085 (47)
University	28 (14)	99 (22)
**Origin, n (%)**		
Spain	137 (69)	2554 (58)
Latin America	23 (11)	726 (16)
Western Europe	32 (16)	773 (17)
Africa	8 (4)	245 (6)
Other	0 (0)	25 (1)
**AIDS diagnosis, n (%)**	66 (33)	790 (18)
**Nadir CD4 + (cells/μL), median (IQR)**	173 (56, 264)	250 (135, 342)
**CD4 + (cells/μL), median (IQR)**	424 (266, 663)	569 (404, 761)
**CD8 + (cells/μL), median (IQR)**	880 (621, 1362)	891 (661, 1198)
**CD4/CD8 ratio**	0.48 (0.28, 0.81)	0.64 (0.42, 0.92)
**ART regimen**		
NNRTI	92 (46)	2631 (60)
PI	90 (45)	1408 (31)
INSTI	9 (5)	248 (6)

Abbreviations: ART, antiretroviral therapy; IDU, injecting drug use; INSTI, integrase strand transfer inhibitor; MSM, men who have sex with men; NNRTI, non-nucleoside reverse transcriptase inhibitor; PI, protease inhibitor.

a Baseline was the visit at month 24 after ART initiation.

Two years after ART initiation, 3599 (78%), 3301 (71%), 2931 (63%), and 2455 (53%) of the participants had a CD4/CD8 ratio ≥0.2, ≥0.3, ≥0.4, and ≥0.5, respectively. Similarly, 3586 (78%), 3099 (67%), 2292 (50%), and 986 (21%) reached a CD4 count ≥200, 350, 500, and 750 cells/μL. Twelve percent were censored because of loss to follow-up (n = 401, 9%) or ART discontinuation (n = 150, 3%).

**Fig. 1:**
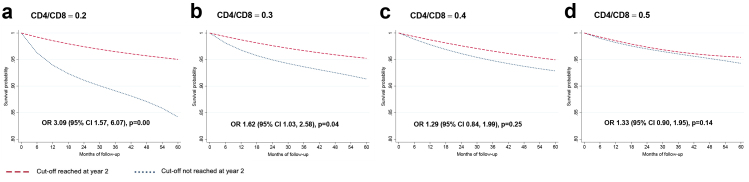
**Survival curves for each CD4/CD8 cut-off**. Survival probability and odds ratio (95% CI) for each subgroup of participants. The outcome studied is the cumulative incidence of serious non-AIDS events. The baseline visit (month 0 of follow-up) corresponds to 24 months after antiretroviral therapy initiation. The OR of presenting a clinical event corresponds to the five-year follow-up period.

**Fig. 2:**
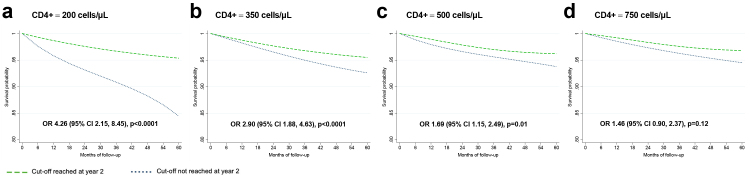
**Survival curves for each CD4+ cut-off**. Survival probability and odds ratio (95% CI) for each subgroup of participants. The outcome studied is the cumulative incidence of serious non-AIDS events. The baseline visit (month 0 of follow-up) corresponds to 24 months after antiretroviral therapy initiation. The OR of presenting a clinical event corresponds to the five-year follow-up period.

**Fig. 3:**
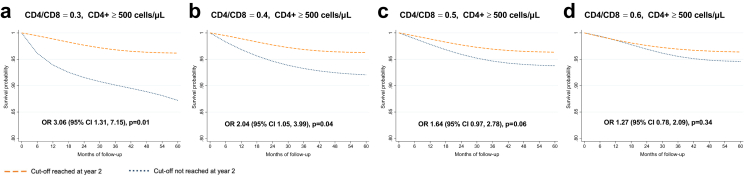
**Survival curves for each CD4/CD8 cut-off among participants with CD4+ count ≥500 cells/μL**. Survival probability and odds ratio (95% CI) for each subgroup of participants. The outcome studied is the cumulative incidence of serious non-AIDS events. The baseline visit (month 0 of follow-up) corresponds to 24 months after antiretroviral therapy initiation. The OR of presenting a clinical event corresponds to the five-year follow-up period.

During the five-year follow-up, 4.3% had a SNAE: 48 (1%) had a MACE, 105 (2.3%) were diagnosed with a NADM, and 47 (1%) had non-accidental death, as shown in Table 2. Figure S2 show the unadjusted estimates (Kaplan–Meier curves and log-rank test). Fig. 1 shows the adjusted survival curves and odds ratios (OR) for the event during follow-up for the CD4/CD8 models. A CD4/CD8 ratio <0.3 at year two of ART predicted an increased risk of SNAEs during follow-up (OR 1.63 [95% CI 1.03, 2.58]). At the lowest CD4/CD8 ratio cut-off (<0.2), the effect became stronger (OR 3.09 [95% CI 1.57, 6.07]). Fig. 2 shows the effect of CD4 count at year two on SNAE risk. A low CD4 count was associated with an increased risk of clinical events at cut-offs of 200, 350, and 500 cells/μL, above which the predictive ability was lost. Among participants with CD4 count ≥500 cells/μL, we found an increased risk of SNAEs at the 0.3 and 0.4 CD4/CD8 cut-offs (Fig. 3). In analyses exploring the CD8 count effects in participants with a CD4 count ≥500 cells/μL, we found that a CD8 count ≥1500 cells/μL, but not the 800 and 1000 cells/μL cut-offs, predicted an increased risk of subsequent SNAEs (Figure S3).

**Table 2:** Outcomes during follow-up.

Event	N (%)
**Nonaccidental death**	47 (23.5)
**MACE**	48 (24)
Myocardial infarction	35 (17.5)
Stroke	13 (6.5)
**NADM**	105 (52.5)
Anus	19 (9.5)
Hodgkin's lymphoma	12 (6)
Skin (non-melanoma)	10 (5)
Lung	8 (4)
Liver	7 (3.5)
Colorectal	6 (3)
Non-specified metastasis	6 (3)
Cervical	5 (2.5)
Hematologic	5 (2.5)
Prostate	5 (2.5)
Soft tissue	4 (2)
Head and neck	4 (2)
Bladder	3 (1.5)
Melanoma	2 (1)
Uterus	2 (1)
Breast	1 (0.5)
Brain	1 (0.5)
Pancreas	1 (0.5)
Penis	1 (0.5)
Stomach	1 (0.5)
Testicle	1 (0.5)
Vulva	1 (0.5)

Abbreviations: MACE, major adverse cardiovascular event; NADM, non-AIDS-defining malignancy.

Subanalyses according to event type yielded consistent estimates for all outcomes (MACE, NADM, or non-accidental death). However, they only reached statistical significance for death and MACE at a CD4/CD8 ratio cut-off of <0.2. The CD4/CD8 ratio was more predictive of death or MACE than NADM (OR 3.52, 95% CI 1.03–12.4; OR 4.4, 95% CI 1.4–12.7, and OR 2.4, 95% CI 0.8–6.9, respectively) (Table S1). The *on-treatment* secondary analysis showed results similar to those of the main analysis (Table S2).

The CD4/CD8 ratio correlated with both the CD4 count (Rho 0.53, p < 0.0001) and CD8 count (Rho −0.47, p < 0.0001). However, among patients with a CD4 count ≥500 cells/μL, the correlation with the CD4 count became weaker (Rho 0.31, p < 0.0001), while that with the CD8 count was stronger (Rho −0.63, p < 0.0001) (Figure S4).

## Discussion

In this prospective multicenter cohort of treatment-naïve PWH, we compared the ability of CD4 count, CD8 count, and CD4/CD8 ratio measured two years after ART to predict the risk of SNAEs and mortality over the subsequent five years. The ratio (<0.3) was the only variable that predicted the risk across the entire range of CD4 counts.

Was the association of the CD4/CD8 ratio with the risk of SNAE driven by CD4 count, CD8 count, or both? Interestingly, both the CD4 and CD8 count contributed to the risk prediction, but the extent of immune suppression influenced their contribution. In patients with a CD4 count <500/μL, the association between a low CD4/CD8 ratio and SNAE risk is mainly driven by CD4 count. Conversely, in patients with a higher CD4 count, the association depends on the CD8 count, consistent with our previous findings in a different cohort.^8^ These results suggest that, in patients with a low CD4 count, much of the risk associated with a low ratio is driven by immunodeficiency. In contrast, the risk associated with a low ratio in those with a higher CD4 count is driven by inflammation and immunosenescence (reflected as a high CD8 count). Both mechanisms seem to be captured by the CD4/CD8 ratio across the entire CD4 spectrum, although the CD4/CD8 ratio predictive capacity becomes stronger as the CD4 count increases.

The most predictive CD4/CD8 ratio and CD8 count cut-off values represent an area of controversy. In this study, the most extreme CD4 count, CD8 count, and CD4/CD8 ratio cut-off values (<200 cells/μL, ≥1500 cells/μL, and <0.2, respectively) predicted the most significant SNAEs risk. However, the association disappeared at values far outside their normal range in the general population (≥500 cells/μL, <1000 cells/μL, and ≥0.5, respectively). While a CD4/CD8 ratio <1 is considered abnormal in the general population^22^ and an independent predictor of mortality,^23^^,^^24^ the finding that the CD4/CD8 ratio cut-off for predicting SNAEs risk in PWH on ART is much lower (0.4) is consistent with some previous studies.^8^^,^^9^^,^^25^^,^^26^ In this regard, it is important to note the differences in age between the different studies published in the general population and the population with HIV, which could lead to confusion, especially when analyzing clinical events. For CD8 count, to the best of our knowledge, no studies have investigated the most discriminative cut-off values. Consistent with our findings, two previous studies have reported that a CD8 count >1500 cells/μL predicted SNAEs. In the Copenhagen cohort, a CD8 count >1500 cells/μL measured after ten years of ART predicted an 80% increased risk of non-AIDS-associated mortality compared to a lower CD8 count.^19^ In the AIDS clinical trials group longitudinal linked randomized trials (ALLRT) cohort, a CD8 count >1500 cells/μL at year two of ART predicted a 75% increased risk of AIDS and non-infectious non-AIDS events during the following five years of treatment.^13^

In addition to the different CD4/CD8 ratios and CD8 counts used in previous literature to define associations with SNAEs risk, another important source of heterogeneity across studies is the timing of CD4 and CD8 measurements relative to ART initiation, including time-varying variables^11^^,^^12^^,^^14^^,^^27^^,^^28^ These variables are less interpretable and more challenging to translate to the clinic.^29^ Furthermore, measurements taken proximal to the event can make the role of the CD4/CD8 as a predictor less clear.^17^^,^^25^^,^^30^ Instead, we used a landmark analysis, that is, we measured our predictor variable at year two from ART initiation. Landmark analysis offers several advantages: (i) Simplicity: the results are more interpretable for clinicians. Our analysis provides information on SNAEs predicted by the CD4/CD8 ratio or CD8 lymphocytes measured at a time when most immune activation markers are in a plateau phase^31^^,^^32^; (ii) Methodological: a time-updated CD4/CD8 ratio would be affected by the CD4 drop known to occur before the development of SNAEs^33^; iii) Consistency and reproducibility with subsequent work: landmark analysis facilitates comparison of the effects across studies^13^^,^^34^^,^^35^; and iv) Time horizon, as this strategy allowed us to predict the mid-term risk of clinical progression (through years 3–7), which will be more informative for clinicians.

The major strength of this study is that, in contrast to other studies, the statistical methodology we followed allowed us to simultaneously control for confounding and possible selection bias due to informative censoring, an issue that should be considered especially in cohort studies with prolonged follow-up. The use of adjusted survival curves overcomes the shortcomings of using the hazard ratio as a measure of effect, which can sometimes be uninformative, as it is a weighted average of the time-specific hazard ratios.^36^ In contrast, survival and risks are presented as depending on time, e.g., the 5-year survival. In addition, we used a robust definition of SNAEs, which were prospectively adjudicated in CoRIS. Specifically, we included MACEs and NADMs, as these are the SNAEs that have demonstrated a more consistent association with the CD4/CD8 ratio in prior research.^7^^,^^9^, ^10^, ^11^, ^12^^,^^25^ With respect to the reported incidences of events, it should be noted that this study only included events that occurred between years three and seven of ART initiation.

Certain limitations of this study must be considered. First, although we used a prospective multicenter cohort with a large sample size and long follow-up, as in any observational study, there is a risk of unmeasured confounding. Some potentially confounding lifestyle-related variables are not collected in the cohort, as is CMV seropositivity. However, this covariate should not represent a significant source of bias in our study, given the high seroprevalence of CMV antibodies in adult PWH.^37^ In addition, this cohort has a small representation of women and certain ethnic groups, as well as INSTI-based first-line ART, the therapy currently recommended in clinical guidelines,^15^^,^^16^ contrary to when the study participants started ART.

We believe that the field needs to unify the criteria for CD4/CD8 and CD8 evaluation, and we propose using landmark analysis, as in this study. This could help in systematic review evaluations and allow external validation of the results. A future direction is to understand whether these altered immune profiles (low CD4/CD8 ratio or high CD8 count) protect against certain non-AIDS events, such as the risk of bacterial infections, as suggested in a previous study.^13^ In addition, it is possible that the CD4/CD8 ratio and CD8 count are linked to some, but not all, types of non-AIDS events. In our study, their association with the risk of non-accidental mortality and MACE was stronger than that with the risk of NADM. The results of this work will help to identify PWH at higher risk of clinical events that require closer monitoring, and also to select the population most susceptible to study strategies to improve CD4/CD8, such as physical activity.^38^ Future studies should investigate which types of events are more strongly associated with the CD4/CD8 ratio and CD8 count, and explore the potential mechanisms.

In summary, the results of this large prospective cohort support the use of a CD4/CD8 ratio cut-off of <0.3, regardless of CD4 count, to identify PWH with an excess risk of cardiovascular events, non-AIDS-defining malignancies, and all-cause mortality. A CD8 count of ≥1500 cells/μL also predicts an increased risk, but the CD4 count does not add prognostic information beyond 500 cells/μL. In addition to identifying PWH that might benefit from closer monitoring, our results encourage the evaluation of the CD4/CD8 ratio and CD8 count as surrogate endpoints for new therapies for PWH.

## Contributors

J.M-S. and S.S-V., conceptualized the study and verified the underlying data; J.M.-S. and S.S.-V. analyzed the data, generated the figures, and wrote the first draft of the manuscript; J.D.-A., M.R., R.R., and S.M. contributed to writing and figures generation; J.M.-S., J.A.I, E.B., F.G., A.R.S., N.C., J.O., S.M., and S.S.-V. contributed to data mining. The CoRIS consortium contributed to data collection and management. All authors revised and approved the final manuscript.

## Data sharing statement

All data analysed in this study can be obtained by a reasonable request to the corresponding authors and the CoRIS cohort committee.

## Declaration of interests

J.M.-S. reports personal fees from ViiV Healthcare, Janssen Cilag, Gilead Sciences, and MSD, non-financial support from ViiV Healthcare, Jannsen Cilag, and Gilead Sciences, and research grants from Gilead Sciences, outside the submitted work. S. S.-V. reports personal fees from Gilead Sciences, MSD, Mikrobiomik, and Aptatargets, non-financial support from ViiV Healthcare and Gilead Sciences, and research grants from MSD and Gilead Sciences, outside the submitted work. S.M. reports grants, personal fees and non-financial support from ViiV Healthcare, personal fees, and non-financial support from Janssen, grants, personal fees and non-financial support from MSD, grants, personal fees, and non-financial support from Gilead, outside the submitted work. F.G. reports personal fees and non-financial support from ViiV Healthcare and Janssen Cilag.
